# The TGF-beta-Pseudoreceptor BAMBI is strongly expressed in COPD lungs and regulated by nontypeable *Haemophilus influenzae*

**DOI:** 10.1186/1465-9921-11-67

**Published:** 2010-05-31

**Authors:** Daniel Drömann, Jan Rupp, Kristina Rohmann, Sinia Osbahr, Artur J Ulmer, Sebastian Marwitz, Kristina Röschmann, Mahdi Abdullah, Holger Schultz, Ekkehard Vollmer, Peter Zabel, Klaus Dalhoff, Torsten Goldmann

**Affiliations:** 1 Medical Clinic III, University of Schleswig-Holstein, Campus Lübeck, 23538 Lübeck, Germany; 2 Institute of Medical Microbiology and Hygiene, University of Schleswig-Holstein, Campus Lübeck, 23538 Lübeck, Germany; 3 Department of Immunology and Cell Biology, Research Center Borstel, 23845 Borstel, Germany; 4 Clinical and Experimental Pathology, Research Center Borstel, 23845 Borstel, Germany; 5 Medical Clinic, Research Center Borstel, 23845 Borstel, Germany

## Abstract

**Background:**

Nontypeable *Haemophilus influenzae *(NTHI) may play a role as an infectious trigger in the pathogenesis of chronic obstructive pulmonary disease (COPD). Few data are available regarding the influence of acute and persistent infection on tissue remodelling and repair factors such as transforming growth factor (TGF)-β.

**Methods:**

NTHI infection in lung tissues obtained from COPD patients and controls was studied *in vivo *and using an *in vitro model*. Infection experiments were performed with two different clinical isolates. Detection of NTHI was done using *in situ *hybridization (ISH) in unstimulated and in *in vitro *infected lung tissue. For characterization of TGF-β signaling molecules a transcriptome array was performed. Expression of the TGF-pseudoreceptor BMP and Activin Membrane-bound Inhibitor (BAMBI) was analyzed using immunohistochemistry (IHC), ISH and PCR. CXC chemokine ligand (CXCL)-8, tumor necrosis factor (TNF)-α and TGF-β expression were evaluated in lung tissue and cell culture using ELISA.

**Results:**

In 38% of COPD patients infection with NTHI was detected *in vivo *in contrast to 0% of controls (p < 0.05). Transcriptome arrays showed no significant changes of TGF-β receptors 1 and 2 and Smad-3 expression, whereas a strong expression of BAMBI with upregulation after *in vitro *infection of COPD lung tissue was demonstrated. BAMBI was expressed ubiquitously on alveolar macrophages (AM) and to a lesser degree on alveolar epithelial cells (AEC). Measurement of cytokine concentrations in lung tissue supernatants revealed a decreased expression of TGF-β (p < 0.05) in combination with a strong proinflammatory response (p < 0.01).

**Conclusions:**

We show for the first time the expression of the TGF pseudoreceptor BAMBI in the human lung, which is upregulated in response to NTHI infection in COPD lung tissue *in vivo *and *in vitro*. The combination of NTHI-mediated induction of proinflammatory cytokines and inhibition of TGF-β expression may influence inflammation induced tissue remodeling.

## Introduction

Pulmonary presence of nontypeable Haemophilus influenzae (NTHI) has been implicated as an important infectious trigger in chronic obstructive pulmonary disease (COPD) [1]. New acquired NTHI strains isolated from patients with exacerbations of COPD appear to be one mechanism underlying recurrent exacerbations of chronic obstructive pulmonary disease since they induce more airway inflammation and likely have differences in virulence compared with colonizing strains [2]. Change in bacterial load alone is unlikely to be an important mechanism for exacerbations [3].

Bacterial infection is not only associated with advanced airway inflammation and increased frequency of exacerbations but also related to accelerated decrease in lung function, which suggests a role of bacterial pathogens in the progression of COPD [4].

The pulmonary inflammatory response is a critical element of the host defense to infection and initiates tissue repair to return the organ to normal function. However, an accurate balance between host defense and inappropriate tissue damage is essential. Under the conditions of repeated cycles of infection this balance is frequently challenged [5].

Inflammation induces subsequent release of repair factors, such as vascular endothelial growth factor, keratinocyte growth factor and transforming growth factor-β (TGF-β). Uncontrolled or prolonged repair function and matrix deposition leads to fibrosis, whereas unopposed tissue destruction can cause damage of the alveolar wall with development of emphysema [6]. TGF-β functions as a central regulator that induces tissue remodeling and repair. In experimental models TGF- signaling is necessary for the induction of fibrosis after inflammatory insults [7]. In addition, TGF-β has important immunomodulating effects [8,9].

To characterize regulation of TGF-β signaling molecules by NTHI infection we performed a transcriptome array in an *ex vivo *infection model of human lung tissue.

One of the genes strongly upregulated upon infection was the TGF-β-pseudoreceptor BMP and activin membrane-bound inhibitor (BAMBI). The BAMBI gene encodes a 260 amino acid transmembrane glycoprotein which is highly evolutionary conserved in vertebrates [10] and is related to the TGF-β family type I receptors. BAMBI is induced by members of the TGF- family and β-catenin [11] and functions as a negative regulator of TGF-β signaling by acting as a pseudoreceptor [12]. A role of BAMBI in lipopolysaccharide mediated hepatic fibrosis has been suggested recently [13]. However expression and function of BAMBI in the lung has not been described up to now.

Due to the central role of TGF-β as a regulator of inflammation and repair the aim of this study was to characterize the expression of BAMBI in the human lung and to investigate the influence of NTHI infection as a common trigger of inflammation in COPD on the regulation of the pseudoreceptor.

NTHI infection was studied *in vitro *using a human lung tissue infection model. Persistent infection was evaluated using lung tissue obtained from COPD patients without evidence of acute infection.

## Subjects and methods

### Study protocol

NTHI infection in lung tissues obtained from COPD patients and controls was studied *ex vivo*. Detection of NTHI was done using nested-PCR and *in situ *hybridization (ISH) in unstimulated and in *ex vivo *infected lung tissue by using an acute NTHI infection model which was previously described using other microorganisms [14,15]. This study was approved by the ethical committee of the University of Lübeck (reference number 03/158) and is in compliance with the Helsinki declaration.

### Lung tissues

Lung tissue preparation was done as previously described [15]. Briefly, the specimens were tumor-free material at least 5 cm away from the tumor front. For *ex vivo *infection experiments lung specimens (1 cm^3 ^size) were cultured in RPMI1640 medium (Sigma, Taufkirchen, Germany) at 37°C and 5% CO_2 _for 24 h and incubated with 500 μl NTHI suspensions (10^7 ^CFU/ml) or medium [14]. Tissues were fixed using the HOPE (Hepes glutamic acid buffer mediated Organic solvent Protection Effect) technique [16]. Viability of tissue was assessed by LDH assay and showed no significant increase during an incubation period of up to 48 h (data not shown).

### Culture and characterization of NTHI

The NTHI strains used in this study were clinical isolates from the University Hospital in Luebeck. Strain 1 (defined as NTHI-1) was an isolate from a COPD patient with invasive, pneumonic disease, whereas strain 2 (defined as NTHI-2) was a noninvasive respiratory isolate from a patient without COPD. Both strains were characterized by biochemical assays (API-NH, Fa. BioMeriéux, Nürtingen, Germany), the requirement of factor × and V for bacterial growth, and negative slide serum agglutination tests. Sequencing of the 16 S rRNA gene region revealed the Rd KW20 NTHI strain in both cases. For the experiments, NTHi were grown overnight on chocolate agar at 37°C and 5% CO_2_. The working solution was adjusted to 1.2 × 10^9 ^bacteria/ml using densitometry.

### Transcriptome array

Total RNA was extracted from HOPE-fixed, paraffin-embedded lung tissues which were *in vitro *infected with NTHI or subjected to medium only [17]. To identify regulation of TGF-β signaling molecules induced by NTHI a 44 k transcriptome array was used (Agilent, Böblingen, Germany, [18]). As a usual procedure with this array format the expression values were quantile-normalized [18]. We compared the log-ratios of expression in infected and not infected lung tissues from the same donors.

### Immunohistochemical staining (IHC)

Primary antibodies (BAMBI; mouse anti human, eBioscience, San Diego, USA; TGF-β, rabbit anti human, Abcam, Cambridge, UK) were applied in a dilution of 1/100 as described elsewhere [19]. Identification of cell types was performed morphologically by lung pathologists and validated by immunohistochemistry using expression of CD68 for macrophages and of SP-A for alveolar epithelial cells type II; Bronchial epithelia were identified by their morphology.

In total we analyzed 48 samples from COPD lungs including 10 samples of *in vitro *infected lung specimens. In addition 11 samples from patients without COPD were analyzed.

### ISH

For targeting NTHI by a specific DNA-probe, a 146 bp (Rd KW20) sequence was amplified using the following primers for: TCG CTG ATT TTC CCG GTT TA, rev: TAG CAA GCA AAG ATT GCT CC fragment was carried out overnight in moist chambers at 46°C. For targeting BAMBI mRNA the following primers were used (Bambi for: CAG CTA CAT CTT CAT CTG GC; Bambi rev: AGA AGT CTA GAG AAG CAG GC), which span an amplicon of 152 bp and were also used for RT-PCR. Probes were generated and hybridized like previously described [14,17]. Sequencing was performed to verify the specificity of the RT-PCR. All samples were analyzed by two independent investigators (TG and DD).

### Real-time polymerase chain reaction (RT-PCR) of Bambi mRNA expression

RT-PCR was performed using NucleoSpin RNA II kit (Macherey-Nagel, Dueren, Germany) and reverse transcribed into cDNA (Roche First- Strand PCR kit, Mannheim, Germany), PCR amplification was performed using LightCycler^® ^Detection System (Roche Molecular Biochemicals, Penzberg, Germany). Conventional RT-PCR was performed as previously reported [17] and the results were normalized to GAPDH.

### Cytokine assays

Measurement of CXC chemokine ligand (CXCL)-8, tumor necrosis factor (TNF)-α and TGF-β levels in supernatants was performed using commercially available ELISA kits (Biosource, Solingen, Germany).

### Western Blot

Lung homogenates and cell pellets were lysed, subjected to 12% SDS-PAGE, and blotted on nitrocellulose membrane (Sartorius, Goettingen, Germany). Immunodetection of phosphorylated p38 MAPK was performed with specific antibodies (Cell Signaling Technology, Beverly, USA).

### Bronchoscopy and isolation of BAL cells

Bronchoscopically guided lavage and isolation of AM was performed as described previously [20].

### Statistical analysis

Data are presented as the mean ± SD. Statistics were performed with non-parametric tests. For independent samples Student's t test was used. For categorical variables 2/2 tables were analysed using chi square test. p values > 0.05 were considered statistically significant. Calculations were carried out with Statistica TM for Windows (version 5), 1997.

## Results

### Patients and lung tissue

The study population consisted of 48 COPD patients (mean age 63 years, 34 males, 14 females) who had an indication for lung surgery of peripheral nodules (table 1). No patient had undergone antimicrobial treatment before the operation. Systemic steroid treatment was administered preoperatively in 13/48 patients in doses < 20 mg/d of prednisone equivalent.

**Table 1:** Demographic data and NTHI detection in lung tissues from COPD patients and controls.

Study group Treatment	n	pack years	NTHI detection		Steroid
			PCR	ISH	
COPD	48		18 (38%)*	18*	
GOLD I	12	62 [28-145]	4 (33%)	4	
GOLD II (n = 8)	18	67 [34-160]	6 (33%)	6	systemic
GOLD III (n = 8)	18	56 [25-120]	8 (44%)	8	inhalative (n = 8) systemic
Controls	11	0	0	0	inhalative (n = 6)

*p < 0.05 compared to controls (Chi square); ISH = *in situ *hybridization, NTHI = nontypeable *Haemophilus influenzae*

11 patients without chronic airway diseases served as controls (mean age 59 years, 6 male, 5 female). Lung tissue samples were obtained from lobectomy or atypical resections (COPD patients: lung cancer: n = 39, metastases of extrapulmonary tumors: n = 6, benign nodules: n = 3; controls: n = 11, lung cancer: n = 2, metastases of extrapulmonary tumors: n = 7, benign nodules: n = 2).

### The patterns of infected cells vary between in vitro and in vivo infection with NTHI

**Figure 1:**
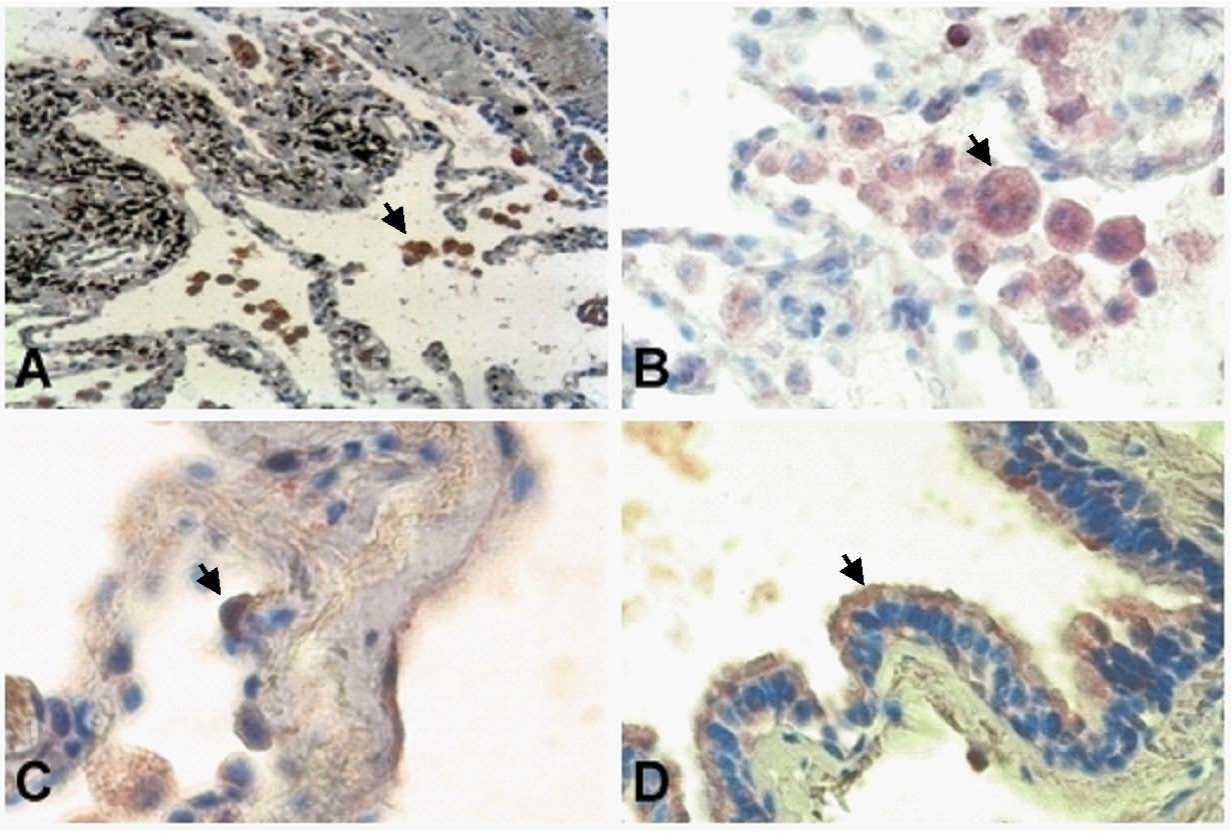
***In situ *hybridization targeting NTHI in persistently infected COPD lung tissue**. AM (A, 400×; B, 600×), AEC (C, 600×) and bronchial epithelial cells (D, 600×). AM = alveolar macrophages, AEC = alveolar epithelial cells. Aminoethylcarbazole was used as a color substrate, which results in red signals. Signals are also indicated by arrows.

**Figure 2:**
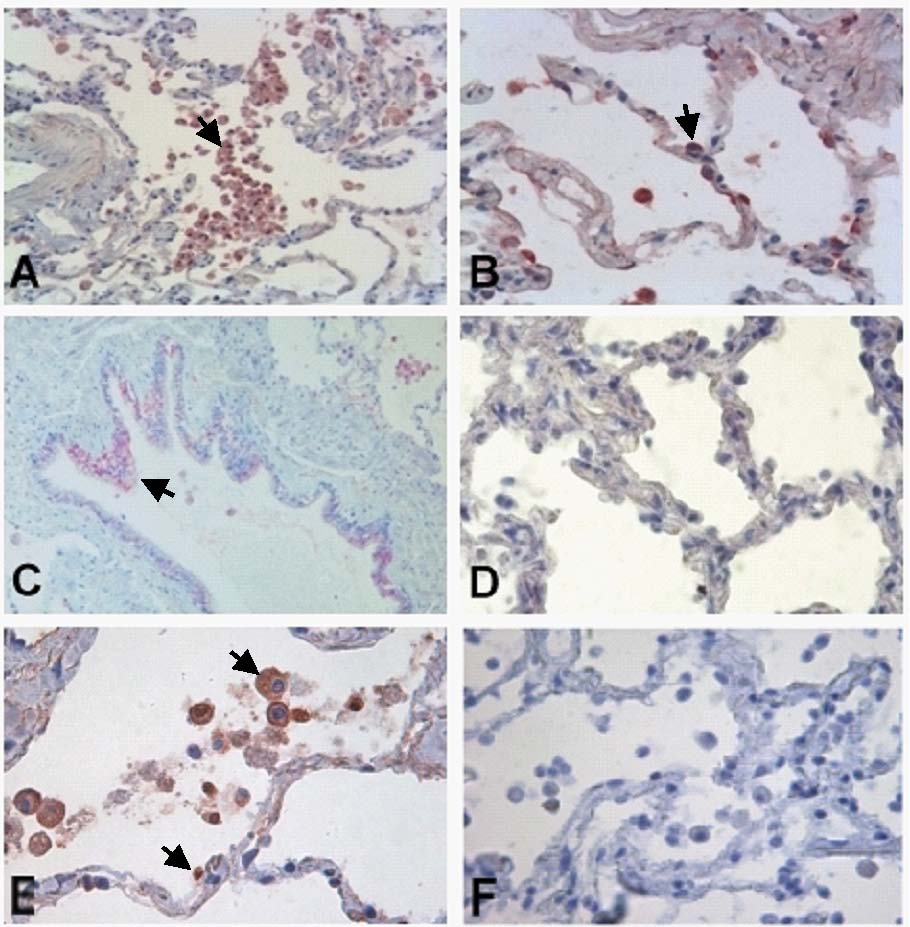
***In situ *hybridization targeting NTHI in *in vitro *infected human lung tissue (Aminoethylcarbazole, red signals)**. Signals are also indicated by arrows. Positive staining of AM (A, 400×), AEC (B, 400×) and bronchial epithelial cells (C, 400×), control (D, 400×). NTHI-1 infection was primarily detected in AM (figure a) and to a lesser degree also in AEC (figure 2b). Similar results were seen with NTHI-2 (demonstrated for AM [E, 600×], control [F, 600×]). AM = alveolar macrophages, AEC = alveolar epithelial cells.

38% (n = 18/48) of COPD lung tissue proved to be NTHI-DNA positive as detected by PCR. Results of the PCR were all confirmed in the ISH. We found no significant differences of infection rates between the different stages of disease (Table 1). NTHI-DNA negative lungs did not show positive signals using ISH. On the cellular level an infection rate (determined by evaluation of positive ISH-staining) of 40-50% in AM and 35-45% in alveolar epithelial cells (AEC) was observed in infected COPD lungs (figure 1). In contrast, after acute *in vitro *infection with strain NTHI-1 and NTHI-2 a different infection pattern was found with infection rates of AM in 60-75% and of AEC in 15-25% (figure 2). In addition, in tissue samples representing bronchial epithelial cells (BEC) we found intense positive staining of these cells targeting NTHI infection *in vivo *and *in vitro *using ISH (figure 1d and 2c). In lung tissues of patients without COPD we did not detect NTHI using both PCR and ISH (p < 0.05, table 1).

### Modulation of TGF-β signalling by infection with NTHI

**Figure 3:**
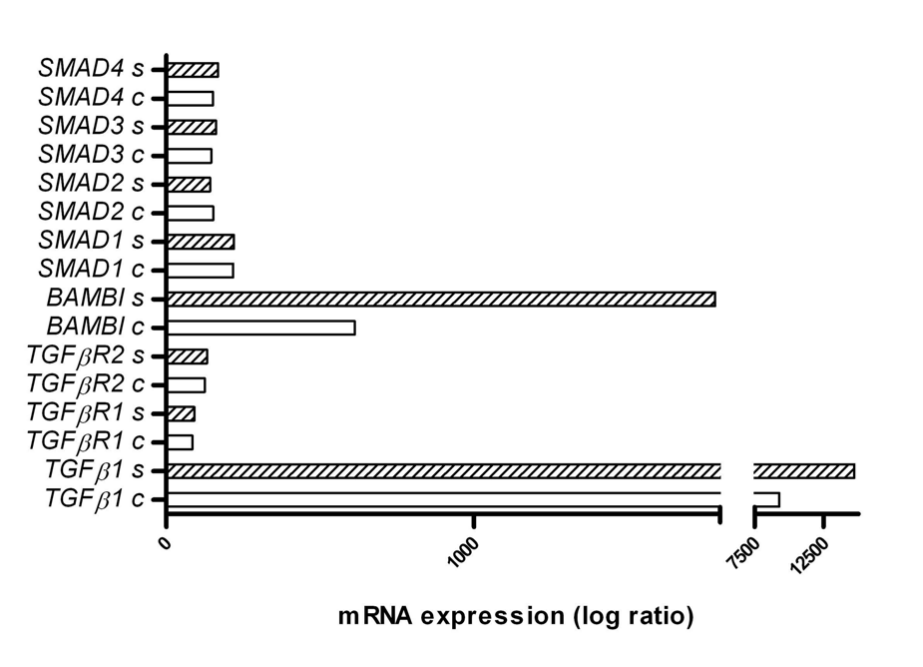
**Transcriptional levels of different molecules involved in TGF-β signalling after NTHI stimulation (C = control, S = NTHI-stimulated) obtained by transcriptome arrays**.

To characterize regulation of TGF-β signaling molecules by NTHI a transcriptome array of *in vitro *infected COPD lung tissue was performed (n = 5). Data of the array showed no significant changes of TGF-β receptors and Smad-3 expression. Regarding TGF-β expression we found a moderate increase, whereas a strong expression of BAMBI with 3-fold increase after *in vitro *infection of COPD lung tissue was demonstrated (figure 3).

### NTHI induced host response

**Figure 4:**
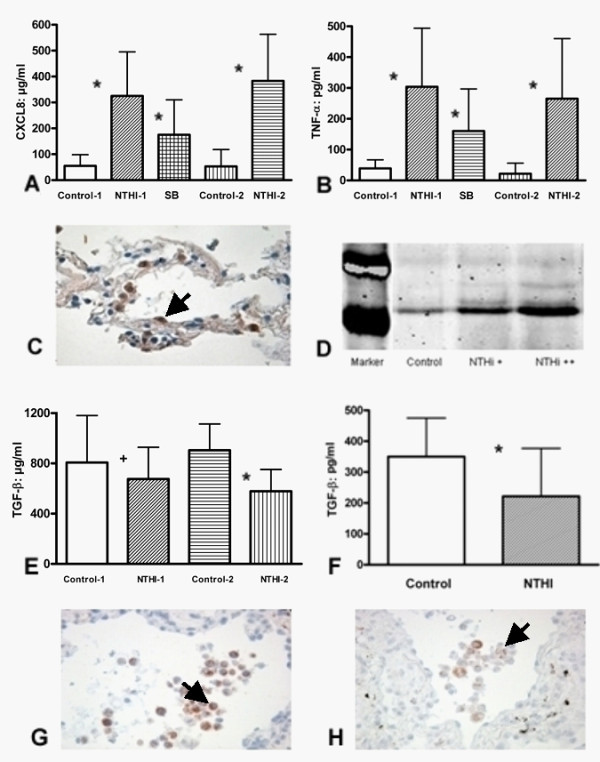
**Expression of CXCL-8 (A) and TNF-α (B) in supernatant of human lung tissue after *in vitro *infection with NTHI-1 and NTHI-2**. Signals are indicated by arrows. Expression of pp38 in human lung tissue (C: IHC, 400×; D: Western Blot). TGF-β expression in supernatant of *in vitro *infected human lung tissue with NTHI-1 and NTHI-2 (E) and A549 cells (F). IHC of TGF-β in human lung tissue without (G, 400×) and with (H, 400×) NTHI-1 *in vitro *infection. SB (203580) = p38 MAPK inhibitor; IHC = Immunohistochemistry, NTHI-1, n = 6; NTHI-2, n = 5; * = p < 0.01, + = p < 0.05.

Measurement of cytokine concentrations from supernatants of *in vitro *infected lung tissue (NTHI-1 and NTHI-2) revealed a strong proinflammatory response with increased expression of CXCL-8 and TNF-α (figure 4a and 4b). Furthermore infection led to increased expression of the MAP-kinase p38, which is demonstrated in figure 4c and 4d. Inhibition of p38 significantly inhibited CXCL-8 and TNF-α expression (p < 0.01). NTHI infection of lung tissues (NTHI-1 and NTHI-2) and A549 cells generated a significant decrease of TGF-β release in the supernatant (p < 0.05 and p < 0.01, figure 4e and 4f). A reduction of TGF-β expression in infected lung tissue was also observed using IHC (figure 4g and 4h).

### BAMBI is strongly upregulated in lung tissue in response to NTHI infection in vivo and in vitro

**Figure 5:**
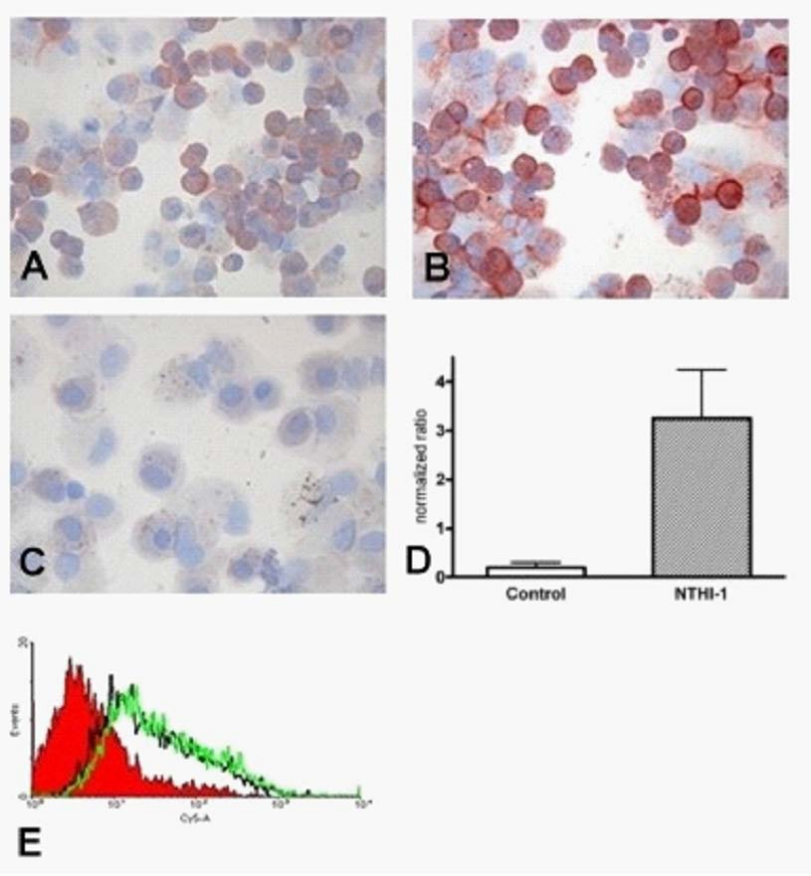
**Expression of BAMBI in isolated AM and alveolar epithelial cells (Aminoethylcarbazole color substrate, results in red signals)**. A: unstimulated AM (600×), B: NTHI-1 stimulated AM (600×), C: control (600×). D: mRNA expression of BAMBI in AM (normalized to GAPDH). E: Expression of Bambi on A549 cells (red area: isotype control). AM = alveolar macrophages. n = 4.

BAMBI was expressed ubiquitously on AM and to a lesser degree on AEC. This was demonstrated using IHC and ISH and was confirmed by RT-PCR and sequencing. Using IHC on isolated AMs a typical membrane-bound pattern is demonstrated (figure 5a and 5b).

**Figure 6:**
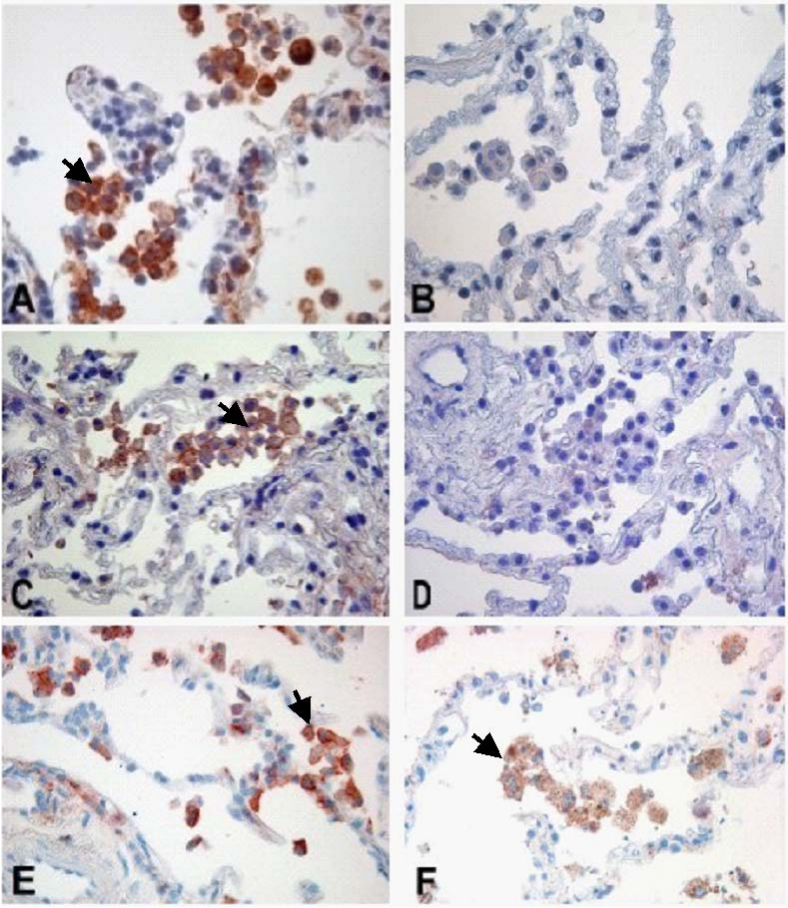
**Protein expression of BAMBI in human lung tissue (IHC, representative samples, red signals are indicated by arrows)**. Induction of BAMBI by *in vitro *infection of NTHI-1 (A, 400×), Medium (B, 400×). Induction of BAMBI by *in vitro *infection of NTHI-2 (C, 400×), Medium (D, 400×). Expression of BAMBI in COPD lung tissue with (E, 400×) and without (F, 400×) NTHI detection. IHC = Immunohistochemistry.

**Figure 7:**
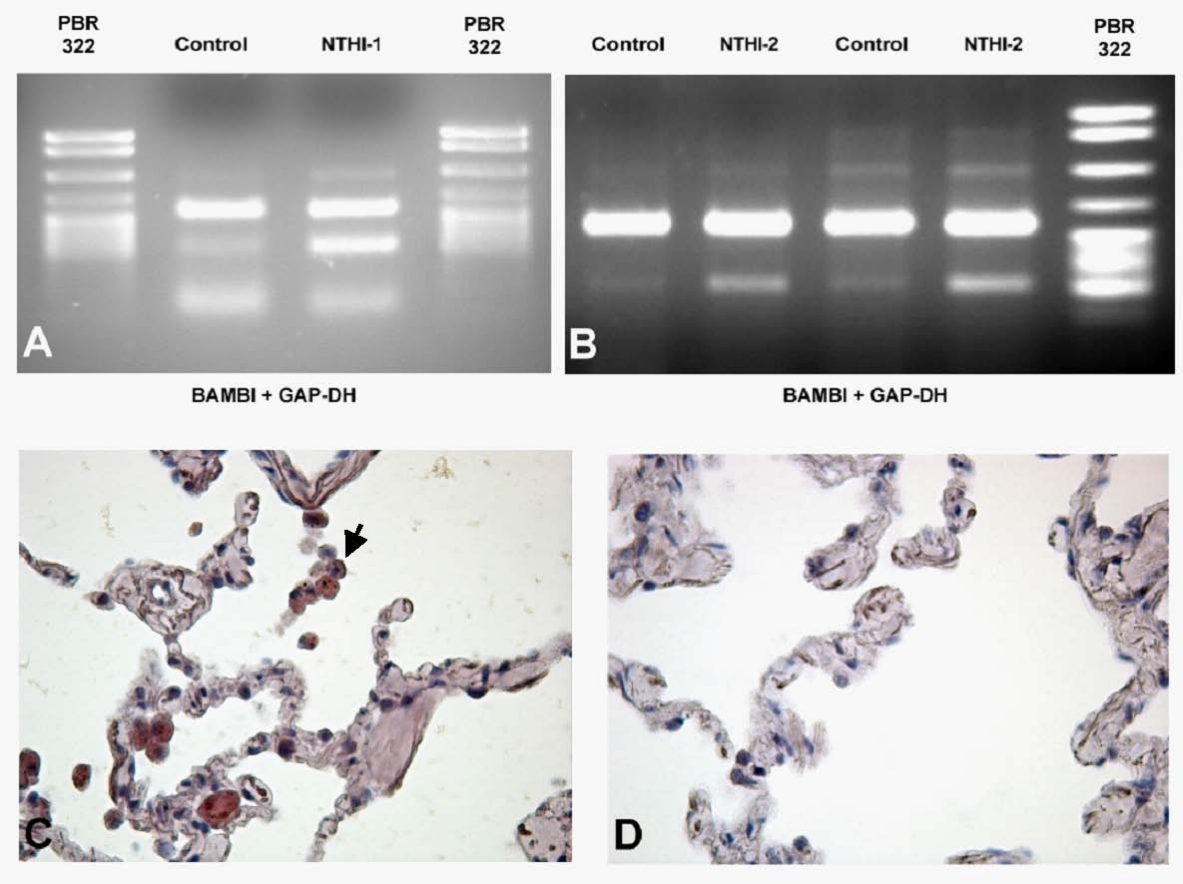
**RNA expression of BAMBI in human lung tissue**. RT-PCR in non infected or *in vitro *infected lungs (upper bands represent GAPDH expression, lower bands BAMBI expression). A: Results for NTHI-1, B: Results for NTHI-2. Expression of BAMBI-RNA in *in vitro *NTHI-1 infected (C, 600×) and non infected (D, 600×) human lung tissue (ISH, red signals, see arrows).

*In vitro *infection revealed that NTHI induces a strong upregulation of BAMBI in the lung tissue on AM and AEC as well as on isolated AM and A549 cells. This was demonstrated on RNA and protein level (figure [5band 5d] and 6[a-d]). This induction was observed uniformly among the different lung tissues, cells and cell lines tested. *In vivo *NTHI-infected lung tissue of COPD patients showed also a stronger expression of BAMBI on AM and AEC compared to lung tissue without NTHI infection and control tissue (figure 6e and 6f). However, there was no correlation with the different GOLD-classes. Figure 7 demonstrates the increased expression of BAMBI on the RNA level in *in vitro *infected lung tissue.

## Discussion

In the present study we demonstrate for the first time the expression of the TGF pseudoreceptor BAMBI in the human lung. COPD patients with NTHI infection showed increased expression of BAMBI in the lung tissues when compared to non-infected patients. Furthermore we show the upregulation of the pseudoreceptor by *in vitro *infection using two different NTHI strains in combination with a strong proinflammatory response and decreased expression of TGF-β.

The characterization of BAMBI adds a new mechanism to the complex regulation of TGF-β in the human lung. Recently the pseudoreceptor, which is able to inhibit TGF-β signaling, was described in the liver, where LPS-induced downregulation of the receptor leads to increased fibrosis [13]. In our study we demonstrate the expression of BAMBI in the human lung with a clearly membranous expression pattern. Signaling of TGF-β is known to be mediated via the TGF receptors I and II which may be prevented by interaction of the cytokine with the pseudoreceptor [12]. This mechanism may influence TGF signalling besides other known activation and signalling pathways [21-25]. Both *in vivo *and acute *in vitro *NTHI infection were associated with marked upregulation of BAMBI. Since TGF-β is a central mediator of tissue rermodeling pathogen induced expression of BAMBI may contribute to impaired tissue repair in COPD. Interestingly NTHI infection of lung tissue and alveolar epithelial cells led to a decreased release of TGF-β which to our knowledge has not been described up to now. This imbalance between expression of pseudoreceptor and cytokine could play a crucial role in TGF-β effector function and may be explained by the binding of TGF-β on BAMBI. In contrast, no significant alteration of TGF receptors I and II in response to NTHI infection was demonstrated. Taken together we speculate that in the lung BAMBI may serve as an inhibitor of excessive TGF-β spillover which could be deleterious for the parenchyma by inducing profibrotic activity.

Smoking may also influence TGF signalling importantly. Acute smoke exposure generates increased TGF-beta levels in animal models [26] which may be due to downregulation of BAMBI induced by LPS from tobacco smoke [27]. In contrast chronic LPS exposure is associated with hyporesponsiveness [28]. This mechanism could explain increased BAMBI expression in chronic smokers with COPD leading to progression of destruction of lung parenchyma. The relative contribution of chronic smoking and/or bacterial infection awaits further study since we were not able to evaluate lung tissue from healthy smokers.

In addition the presence of mediators released by malignant cells or effects of steroid treatment in the lung tissues analyzed here may also have influenced our findings. However, the fact that cell culture experiments using A549 cells generated similar results makes this possibility unlikely.

Bacterial infections are a major cause of exacerbations with NTHI being the most frequent pathogen isolated [29]. We have shown that NTHI is expressed intracellularly in 38% of COPD lungs from patients without evidence for acute exacerbation corresponding to data from a previous study reporting a detection rate of 50% in a group of COPD patients undergoing lung transplantation [30]. We observed that acute *in vitro *NTHI infection leads to a strong proinflammatory cytokine expression, but a reduced expression of TGF-β.

Considering the immunosuppressive properties of TGF-β [8] impaired TGF signalling may contribute to the increased pathogen induced inflammatory response in COPD [31]. On the other hand this imbalance carries the risk of tissue destruction.

In conclusion we observed that the TGF pseudoreceptor BAMBI is expressed and regulated by NTHI in the human lung. This finding may be important for the understanding of inflammatory mechanisms and remodeling in COPD patients. The combination of enhanced proinflammatory cytokine response and impaired repair mechanisms could contribute to the development of lung emphysema. The development of new, more targeted therapeutic approaches [32] requires an even better understanding of the mechanisms of host-pathogen interaction in COPD.

## Competing interests

The authors declare that they have no competing interests.

## Authors' contributions

DD wrote the manuscript. JR provided the NTHI for *ex vivo *experiments. KR and SO performed the ELISA. AJU and KR did the cell culture and BAL analyses. SM and MA did the IHC and RT-PCR. HS and EV took the pathologic part of the study. PZ and KD were involved in the design of the study and in writing the manuscript. TG conceived of the study and conducted the experiments and writing. All authors have read and approved the final manuscript
